# Study of the tensile properties of individual multicellular fibres generated by *Bacillus subtilis*

**DOI:** 10.1038/srep46052

**Published:** 2017-04-05

**Authors:** Xuan Ye, Liang Zhao, Jiecun Liang, Xide Li, Guo-Qiang Chen

**Affiliations:** 1 Department of Engineering Mechanics, AML, Tsinghua University, Beijing 100084, China; 2 Center for Nano and Micro Mechanics, Tsinghua University, Beijing 100084, China; 3 Center for Synthetic and Systems Biology, Tsinghua University, Beijing 100084, China

## Abstract

Multicellular fibres formed by *Bacillus subtilis (B. subtilis*) are attracting interest because of their potential application as degradable biomaterials. However, mechanical properties of individual fibres remain unknown because of their small dimensions. Herein, a new approach is developed to investigate the tensile properties of individual fibres with an average diameter of 0.7 μm and a length range of 25.7–254.3 μm. Variations in the tensile strengths of fibres are found to be the result of variable interactions among pairs of microbial cells known as septa. Using Weibull weakest-link model to study this mechanical variability, we predict the length effect of the sample. Moreover, the mechanical properties of fibres are found to depend highly on relative humidity (RH), with a brittle–ductile transition occurring around RH = 45%. The elastic modulus is 5.8 GPa in the brittle state, while decreases to 62.2 MPa in the ductile state. The properties of fibres are investigated by using a spring model (RH < 45%) for its elastic behaviour, and the Kelvin–Voigt model (RH > 45%) for the time-dependent response. Loading-unloading experiments and numerical calculations demonstrate that necking instability comes from structural changes (septa) and viscoelasticity dominates the deformation of fibres at high RH.

Currently, biologists and material scientists fabricate new biological fibres as substitute for natural or chemically synthesized ones employed in daily or engineering applications. In particular, it is desired for these substitute materials to be non-toxic, pollution-free, and biodegradable, characteristics which are absent in most chemically synthesized fibres. Peptidoglycan, a macromolecular compound combining the features of some natural fibres^1^,^2^, is a main component of bacterial cell walls^3^,^4^,^5^,^6^. For example, *B. subtilis* mutants with deleted genes related to peptidoglycan hydrolase exhibit a filamentous or fibrous state^7^ that can easily form a visible multicellular fibre from a liquid culture^8^,^9^. However, the individual multicellular fibres of various bacteria have varying thicknesses and structures depending on bacterial walls^10^,^11^, as well as on crosslinking degrees of the peptidoglycan. All these affect mechanical properties of multicellular fibres. Therefore, it is important to understand the deformation behaviour of the individual multicellular fibre to further improve the fibre performances for appropriate applications.

To thoroughly understand the mechanical properties of individual *B. subtilis* multicellular fibres, direct experimental measurement is desired. However, currently there are only very few methods able to measure the Young’s modulus of intact peptidoglycan fragments and bacterial cells. For example, quantitative information on sample elasticity (e.g., Young’s modulus, average spring constant, etc.) has been obtained by combining the force–penetration curves obtained via atomic force microscopy (AFM) with nanoindentation theory^12^,^13^,^14^. Also, the viscoelastic response of an individual bacterial cell has been obtained with AFM indention and combined with a cell mechanics model to understand the complex relationship between the cell structure and function^15^,^16^,^17^. AFM provides information with very high spatial resolution, but only measures the properties of the cell wall. Further, such AFM results are difficult to confirm because of the inability to precisely observe the indent process and the complex interaction between the AFM tip and cell wall at the micro- or nanoscale.

Recently, some appealing approaches to study the mechanical properties of bacteria have been proposed. For instance, Tuson *et al*.^18^ provided an experimental approach to measure the mechanical properties of bacteria *in vivo*, which they call “cell length analysis of mechanical properties” (CLAMP). This technique measures the growth rate of an individual bacteria encapsulated in agarose with a user-defined stiffness. Amir *et al*.^19^ applied a controllable hydrodynamic force to bend growing rod-shaped cells of *Escherichia coli (E. coli*) and *B. subtilis* to obtain the mechanical stresses regulating the growth of their cell walls.

Unfortunately, these methods involving AFM, CLAMP, and hydrodynamic force focus on studying the mechanical properties of the cell walls or membranes of a single cell, making them unsuitable for mechanical testing of individual multicellular fibres containing several to hundreds of mutant cells generated by suppression of cell division. Early in 1985, however, Thwaites *et al*. stretched bacterial threads and measured the Young’s modulus of the cell wall as well as other mechanical properties of the bacterial cells^9^,^20^,^21^. The threads in their study contained 20,000 parallel fibres from division-suppressed mutant of *B. subtilis*^22^,^23^. However, their test results for these bacterial threads are not suitable for predicting the mechanical properties of the cell wall and individual cellular fibres. It remains a challenge to manipulate and measure the properties of individual fibres quantitatively because of their nanoscale diameter and extremely high slenderness ratio.

In the present paper, we develop a method for studying the mechanical properties of multicellular fibres generated from cell-separation-suppressed mutants of *B. subtilis* with various genes deleted. To our knowledge, this is the first time that the mechanical properties of cellular fibres with slenderness ratios of 37–363 (i.e., several to several hundred bacterial cells) have been directly measured. These measurements are conducted using a novel manipulation procedure called “liquid drop method (LDM)” on the proposed multilevel measurement platform. Based on these measurements, we study the tensile strength variability using Weibull weakest-link model and present the length effect of the sample. Moreover, we find that the mechanical properties of fibres depend highly on the relative humidity (RH), identify the RH of the brittle–ductile transition of the fibres, and reveal their deformation and failure mechanisms. This study should provide a possibility to understand the deformation mechanisms of bacterial fibres for further improvements on their performance.

## Results and Discussion

### Tensile testing of individual fibres of *B. subtilis* mutants with various genes deleted

The proposed approach and platform under an optical microscope (OM) was used to test individual *B. subtilis* multicellular fibres with various genes deleted. Because the length of a fibre is controlled by various genes related to cell division, we could generate multicellular fibres by deleting *sigD*, a sigma factor that controls the gene expression of peptidoglycan hydrolase including *lytF, lytC*, and *lytD*, and some major peptidoglycan hydrolases such as *lytE* and *lytD*^7^,^24^,^25^.

**Figure 1:**
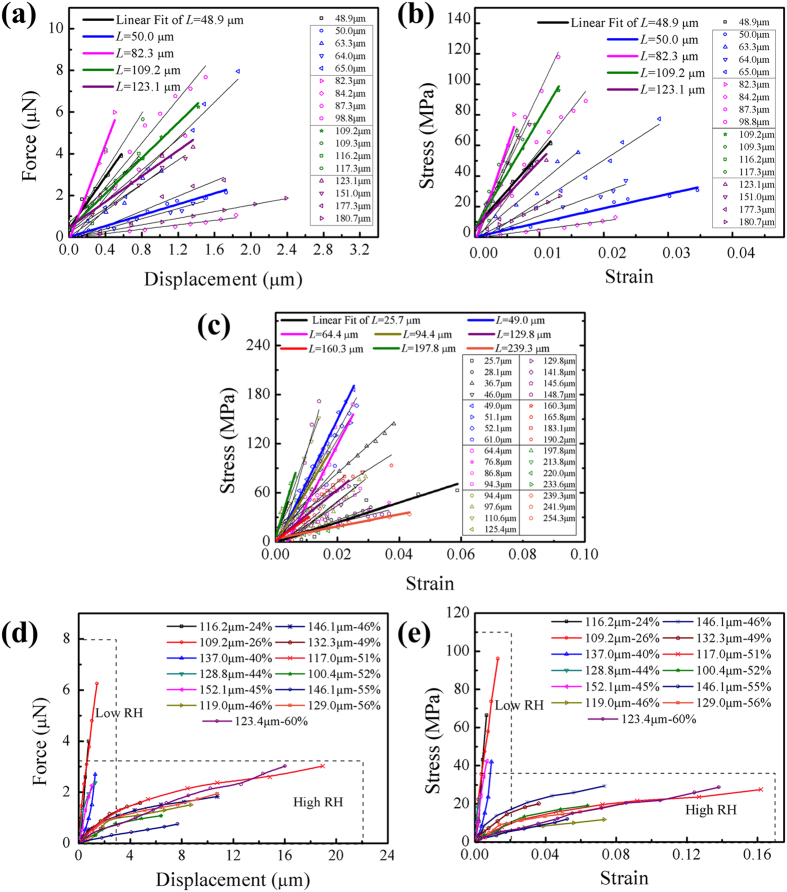
Tensile tests under various humidity levels on *B. subtilis* multicellular fibres with different genes deleted. (**a**) Force–displacement curves of *B. subtilis* multicellular fibres with *sigD, lytE*, and *lytD* deleted. These studies were conducted at 22 ± 2 °C with relative humidity RH = 28 ± 8%. (**b**) Stress–strain curves corresponding to (**a**). (**c**) Stress–strain curves of *B. subtilis* multicellular fibres with *sigD* and *lytE* deleted. These studies were conducted at room temperature with low RH (<45%). (**d**,**e**) Tensile tests of *B. subtilis* multicellular fibres with *sigD, lytE*, and *lytD* deleted under various RH (24–60%). When RH < 45%, the stress and strain have an approximately linear relation, which is a typical property of elastic–brittle materials. When RH > 45%, the stress–strain curves exhibit viscoelastic behaviour and necking failure.

The fibres generated by deleting *sigD, lytE*, and *lytD* were tested at room temperature and RH = 28 ± 8%, where 17 fibres were stretched to fracture. The relationship between force and displacement is approximately linear for the fibres with lengths of 48.9–180.7 μm, suggesting elastic–brittle behaviour (Fig. 1a). The ranges of the fracture force and breaking strain were 1.1–7.7 μN and 0.7–3.6%, respectively, with an average fracture force of 4.5 μN and an average breaking strain of 1.5% (Fig. 1a). To obtain the fracture stress and elastic modulus of the fibres, the force–displacement curves were converted into stress–strain curves, which we accomplished by observing the fibre cross-sections through scanning electron microscopy (SEM). Taking into account the special biological structure of the fibre, we assume it is a hollow cylinder with thickness *t.* Thus, the cross-section *S* of a fibre is 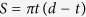, where *d* is the fibre diameter, and the average thickness *t* of the *B. subtilis* cell wall is 40 nm^9^,^10^,^26^. By introducing *S*, the stress–strain curves corresponding to Fig. 1a are plotted in Fig. 1b. These curves produce an average elastic modulus of 5.8 GPa and an average fracture stress of 62.2 MPa with a range of 13.1–117.8 MPa.

We also studied the tensile responses of fibres with *sigD* and *lytE* genes deleted, testing 31 samples with gauge lengths of 25.7–254.3 μm (Fig. 1c). These experiments were conducted at room temperature and RH < 45%. The mechanical responses of these fibres were similar to the results in Fig. 1b, where elastic deformation and brittle fracture appeared at low RH. The ranges of fracture force and breaking strain were 1.3–12.1 μN and 0.4–5.9%, respectively, with an average fracture force of 5.4 μN and an average breaking strain of 2.4%. Further, the average fracture stress and elastic modulus were 82.7 MPa and 4.5 GPa, respectively. When comparing the tensile properties of multicellular fibres with different genes deleted (Table 1), it is found that the elastic modulus of the multicellular fibres is on the same order of magnitude as those of *Bombyx mori (B. mori*) silk, coir, jute, and cotton^27^,^28^,^29^, yet the multicellular fibres have much lower breaking strain at failure and tensile strength.

### Tensile testing of individual *B. subtilis* multicellular fibres under various humidity levels

Previous studies have indicated that humidity affects mechanical properties of cell walls and bacterial threads^17^,^20^,^21^. Figure 1d and e plot the tensile results of *B. subtilis* multicellular fibres with *sigD, lytE*, and *lytD* genes deleted at various humidity levels. Similar to the results in Fig. 1a and b, the force and the displacement (or stress–strain) have an approximately linear relation at low RH (<45%). In this RH range, the maximum fracture force is 6.3 μN and the breaking strain decreases to less than 2.0%. Thus, under dry condition the fibre behaves like an elastic–brittle material with a high average tensile strength (~62.2 MPa) and average elastic modulus (~5.8 GPa). With increasing RH, both the strength and the stiffness decrease, and there is a distinct transition around RH = 45%. As the RH increases from 26 to 51%, the maximum failure force decreases from 6.3 to 3.0 μN and the breaking strain increases from 1.3 to 16.2%. Further, the tensile strength decreases from 96.1 to 27.5 MPa. In this RH range, the shape of the force–displacement (or stress–strain) curves changes to typical viscoelastic behaviour.

**Figure 2:**
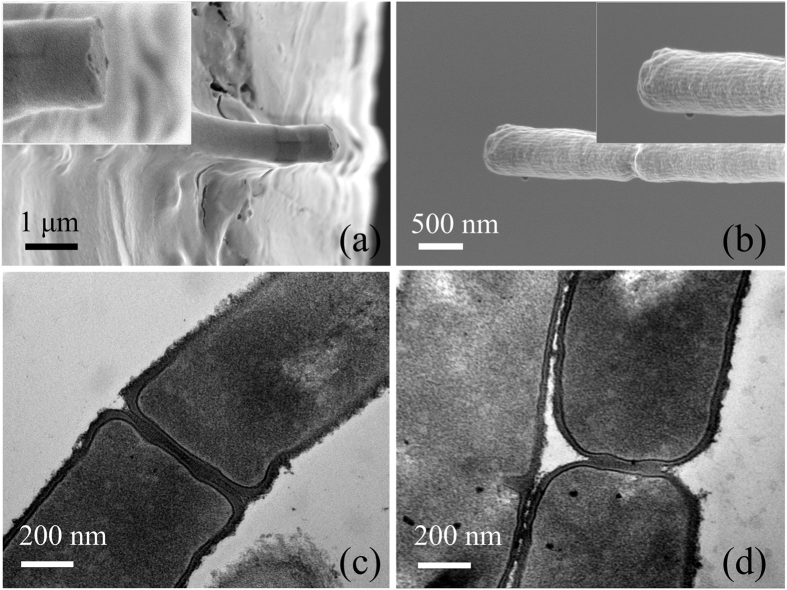
(**a**,**b**) Scanning electron micrographs of the fracture cross-sections of multicellular fibres at low RH. Fracture occurs at the positions of the septa. (**c**,**d**) Transmission electron micrographs of septa at various separation levels.

**Figure 3:**
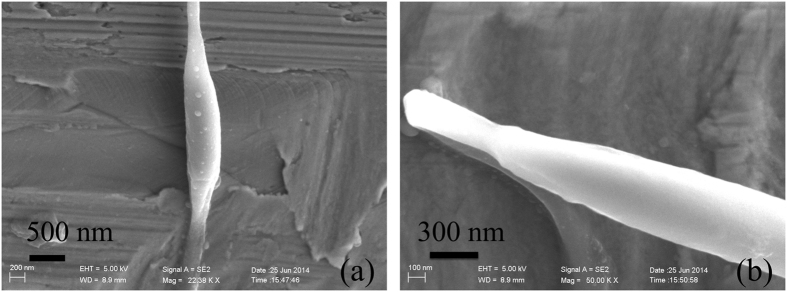
Scanning electron micrographs show ductile deformation of typical multicellular fibres at high RH. Viscoelasticity gradually dominates deformation of the fibres at high RH. The fibre elongates significantly both in the bacterial cells and at the positions of septa, and necking failure occurs.

The SEM images show tensile fracture of fibres at low RH (Fig. 2a and b) and ductile deformation at high RH (Fig. 3). At low RH, the fracture occurs only at the positions of the septa (Fig. 2a and b), contrasting with transmission electron microscopy (H-7650B, Hitachi, Japan) images of the sections of the septa (Fig. 2c and d). However, viscoelasticity dominates deformation of the fibres at RH > 45%. The fibres show significant elongation both in the part of the cell wall and at the positions of septa, and ultimately they neck at the positions of septa.

### Analytical model of individual fibres under tensile loading

Tensile testing reveals that the fibres exhibit different mechanical behaviour under wet and dry conditions (Fig. 1d and e). The fibres show elastic–brittle behaviour under a dry condition, and viscoelastic behaviour under a wet condition.

#### Structure of a B. subtilis cell and its multicellular fibre

*B. subtilis* is a typical gram-positive cylinder-shaped bacterium which has a cell membrane and a cell wall consisting of a porous network of highly cross-linked peptidoglycan attached with teichoic acid^17^. The elastic response of a cell is dominated by the physical structure of the peptidoglycan network and its association with the plasma membrane^16^. The main deformation mechanism of peptidoglycan is a combination of chain unfolding and disentangling. For the peptidoglycan of a *B. subtilis* cell, the rigid glycan strands are mainly oriented in the hoop direction of the cell cylinder, and cross-linked by flexible tetrapeptide molecules. When the cell is stretched parallel to the axis of the cylinder (the long axis of the cell), the peptide chains unfold^11^,^20^,^30^,^31^. Another important component, that is, the plasma membrane, contributes to the viscoelastic response of the cell, however. At the nanoscale, the membrane bilayers exhibit inertial and viscous properties because the constituent lipids and proteins are moving constantly. These special liquid-like properties prevent deformation in the presence of an external force, leading to delayed elastic deformations^16^.

In addition to their cell wall, the septa^30^,^31^,^32^,^33^ in multicellular fibres can play an important role in determining their mechanical properties. An isolated septum has a ridge, ~31 ± 12 nm thick, at its cylinder junction that contains apparent spiral cabling, ~135 ± 40 nm wide, toward the centre. A mature septum may have up to three cables across its radius^31^. However, the average thickness of the cell wall is ~40 nm, and there are 50-nm-wide cables running almost parallel to the short axis of a cell^11^,^31^.

#### Mechanical analysis of B. subtilis multicellular fibres

The mechanical behaviour of the cell envelope is traditionally modelled using mechanical analogues, which consist of a network of elastic springs and viscous dashpots^15^,^16^,^17^. The springs represent the elastic response of the material, and the dashpots describe the fluid behaviour, or the removal and re-establishment of transient bonds.

**Figure 4:**
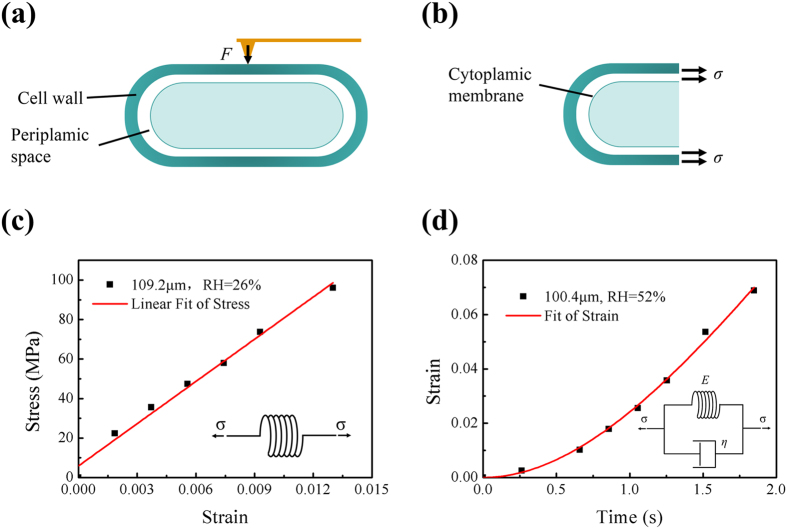
Mechanical models for *B. subtilis* multicellular fibres at low and high RH. (**a**) Schematic diagram of cellular indention developed in previous studies^15^,^16^. The AFM tip first approaches the outer layer of the cell envelope in the short-axis direction, and then transfers the pressure load from the outer layer to the inner layer. (**b**) Tensile test of individual fibre in this study. The load is along the long-axis direction of the *B. subtilis* cell. The cell wall and plasma membrane are simultaneously stretched. (**c**) Spring model is used to describe the elastic properties of the fibre at low RH; this shows a stress–strain curve of a typical sample and the corresponding linear fitting result at RH = 26%. (**d**) Kelvin–Voigt model is used to describe the tensile mechanical behaviour of the fibre at high RH; this shows the parameter fitting for a typical sample at RH = 52%. In the inset, the model is shown as a parallel combination of a spring and a dashpot.

Previous studies have employed an AFM in contact mode to measure local mechanical properties of the cell envelope^15^,^16^. During indention in this method, the AFM tip first approaches the outer layer of the *B. subtilis* cell envelope in the short axis direction, then transfers this pressure load from the outer layer to the inner layer (Fig. 4a). The elastic response of the cell is largely dominated by the peptidoglycan network, and the viscoelastic response of the cell is determined by the membrane^17^. Then, the standard solid model is typically used to interpret the viscoelastic properties of the cell envelope in the short axis direction^15^,^16^,^17^.

For our tensile tests of individual multicellular fibres, the stretching load is along the long axis direction of the *B. subtilis* cell (Fig. 4b), and the septum between the two cells strongly affects fibre’s deformation and especially failure behaviour. Therefore, two mechanical models were employed to describe the tensile behaviour of the fibre: an elastic spring for low RH, and a Kelvin–Voigt model for high RH.

At low RH, there is a high density of hydrogen bonds between peptides in the peptidoglycan of the *B. subtilis* multicellular fibre, producing a very stiff network^22^ and thus increasing the elastic modulus. In this case, the contribution of the plasma membrane is relatively weak, wherein the fibre mainly exhibits elastic behaviour and does not exhibit viscous properties. We represent the elastic properties of the fibre using a spring model (inset in Fig. 4c). For a typical sample with a length of 109.2 μm and a diameter of 558 nm, the fracture stress is 96.1 MPa. The least-squares method fits the stress–strain curve very well, giving an elastic modulus of 7.1 GPa (Fig. 4c).

At high RH (>45%), both the peptidoglycan network and the cell membrane affect the mechanical properties of the fibre^17^,^22^. Because peptidoglycan is elastic and much more flexible than the cell membrane, we use a relatively soft elastic spring to describe the instantaneous elastic deformation of the peptidoglycan network. The cell membrane is viscoelastic, so we use a parallel combination of a spring and a dashpot to describe its delayed elastic deformation. Although the stiffness of the septa is relatively large because of their rigid structure, the septa are weak links. Therefore, we used the Kelvin–Voigt model to describe the tensile behaviour of the fibre at high RH (inset in Fig. 4d). During the tensile tests, we linearly increased the tensile load [refer to equation (1)], and we obtained the strain relationships as follows.


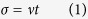



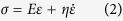


Solving equations (1) and (2), we have


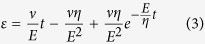


For a typical fibre sample with a diameter of 555 nm and a length of 100.4 μm at RH = 52%, the stress loading rate is *ν* = 7.5 MPa/s and the best fitting parameters are *E* = 63.7 MPa and *η* = 136.0 MPa s (Fig. 4d). This elastic modulus is comparable to that of regenerated *B. mori* silk fibres tested in water, but it is much lower than those of other natural fibres^34^.

Table 2 presents the *E* and *η* of the tested fibres at various humidity levels. At RH > 45%, the fibre is clearly a viscoelastic material with an average elastic modulus of 64 ± 15 MPa and an average viscosity of 110 ± 60 MPas. Compared with the experimental results at RH < 45%, this elastic modulus is two orders of magnitude lower.

Considering that the multicellular fibres are chain-like structures similar to continuous cylinders with invagination at the positions of septa at varying separation levels, the above theoretical model cannot fully characterise how the septa affect deformation. In particular, it is not sufficient to determine that the deformation behaviour of the *B. subtilis* multicellular fibre is dominated only by the viscoelastic mechanism from the stress–strain curves (Fig. 1e) at relatively high RH. Therefore, we also investigate the tensile deformation of multicellular fibres with viscoelastic constitutive relation by finite element analysis (FEA) (see supplementary information for details), respectively. Supplementary Fig. S3 shows fibrous models for the septa at three typical separation levels. The calculated results show that the periodic necking instability occurs at the positions of septa and the deformation mode is similar to that observed under SEM (Supplementary Fig. S5). The viscoelastic constitutive relation can well describe the experimental results, which are all in the range of calculated results with the FEA (Supplementary Fig. S6). To confirm the true deformation mechanism of the tested *B. subtilis* multicellular fibre at relatively high humidity, we performed loading and unloading experiments; Supplementary Fig. S1 shows a loading and unloading curve with typical viscoelasticity. The fibre recovers to its initial length after unloading, and then, a hysteresis loop is also formed. Thus, the periodic necking instability comes from structural changes (septa), and viscoelasticity dominates deformation behaviour of the *B. subtilis* multicellular fibres at high RH.

### Effect of *B. subtilis* multicellular fibre length on tensile properties

**Figure 5:**
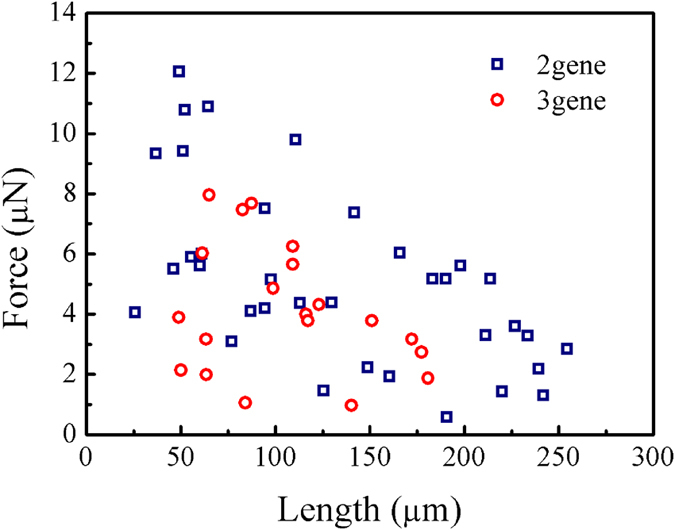
Relationship between fracture load and sample length of multicellular fibres generated from *B. subtilis* with *sigD* and *lytE* genes, and with *sigD, lytE* and *lytD* genes deleted, measured at room temperature and low RH (<45%).

Because of the micro-manipulation procedure and multilevel design of our measurement platform, we can measure the mechanical properties of *B. subtilis* multicellular fibres with various lengths comprising several to several hundred bacterial cells. Thirty-one testing results were acquired for samples with *sigD* and *lytE* genes deleted at RH < 45%, producing relations between the fracture load and sample length (Fig. 5). The tensile strength of the multicellular fibres clearly depends on length. Specifically, the fracture load rapidly decreases with increasing fibre length. For example, a fibre with a length of 49.0 μm has a fracture load of 12.1 μN, while another with a length of 254.3 μm has a fracture load of only 2.8 μN. For multicellular fibres with *sigD, lytE*, and *lytD* genes deleted (∆*SED*), their strength decreases with increasing length as well, but their average strength at lengths of 50.0–180.0 μm is 62.2 MPa. This strength is much lower than the strength of 99.7 MPa of the multicellular fibres with only *sigD* and *lytE* genes deleted (∆*SE*), revealing that deleting the *lytD* gene introduces structural imperfections into the fibres.

### Effect of relative humidity on *B. subtilis* multicellular fibres

Our test results indicate that the RH affects the mechanical properties of *B. subtilis* multicellular fibres with either two or three genes deleted (Fig. 1d). The fibres exhibit almost brittle fracture at RH < 45%, while both their failure force and stiffness progressively decrease with RH when RH > 45%. Additionally, our results show that the fibre transition from brittle to ductile behaviour occurs around RH = 45%. Previous studies have revealed that the elastic modulus and tensile strength decrease with increasing humidity or by immersing samples in water^22^,^35^. The mechanism for this brittle–ductile transition comes from the presence of many hydrogen bonds between the peptides of peptidoglycan in the fibre, which make the polymer networks rigid under dry conditions. As the degree of hydration increases, water competes for the hydrogen bond sites, which makes the networks more flexible. This transition decreases the elastic modulus and strength of the fibres and increases the strain. In addition, water is associated with accessory polymers containing ionisable and hydrophilic groups^22^,^23^,^31^.

The mechanical properties of fibres are sensitive to environmental humidity, which mainly comes from the increased formation of hydrogen bonds in the peptidoglycan under low humidity. This feature has both a negative and positive: On the one hand, a significant change in mechanical properties with varying humidity is clearly unfavourable in structural materials. On the other hand, the deformation characteristics of such environmentally friendly materials can be changed and controlled by changing the humidity, a feature which may have potential applications. For example, such materials could be used for bacterial templating of fibre composites with hierarchical structure^36^,^37^,^38^,^39^, or as buffer packaging materials similar to pulp moulding because of their light weight, easy decomposability, and good ventilation. The humidity-regulated ductile–brittle transition allows the fibres to fit space and shape requirements of templating and packaging at high humidity, while at low humidity they can be used as high-strength support and protection materials.

No significant changes in the dimensions of multicellular fibres appeared at high humidity, but this came from the limitations in the spatial resolution of the OM. Presumably, the lengths and thicknesses of multicellular fibres will increase with increasing humidity because the fibres absorb water and then swell^21^. However, an effective measurement method is still needed to prove this hypothesis. Although SEM can observe changes in the fibre dimensions, the vacuum sample preparation for SEM removes moisture contained in the fibres before observation.

### Genetic effect on *B. subtilis* multicellular fibres

The genes deleted in the present study affect the expression of some peptidoglycan hydrolases. Peptidoglycan is the major structural component of the cell wall of *B. subtilis*, and its synthesis, modification, and hydrolysis are in a dynamic equilibrium associated with bacterial growth and division^31^. There are approximately 30 genes responsible for enzymatic degradation of peptidoglycan^7^,^24^,^25^, of which the genes *lytE, lytF, lytC*, and *lytD* express enzymes with high activities to hydrolyse peptidoglycan with synergic effects. The gene *sigD* is a regulation factor that fully controls the expression of *lytF* and partially controls the expression of *lytE, lytC*, and *lytD*. Thus, as more and more genes related to peptidoglycan hydrolases are deleted, less of the related enzymes will be synthesized. This change reduces the degradation of peptidoglycan and decreases the hydrolysis of the septa between two neighboring bacterial cells, better preserving the cellular structure of the fibre, and making the fibre longer and more stable. This behaviour means that the five genes mentioned in the present study have a synergic effect in controlling the mechanical properties of these multicellular fibres.

Even so, the average fracture force of Δ*SED* is slightly less than that of Δ*SE* (Table 1). As mentioned above, the positions of septa are the multicellular fibres’ weak points where the failure will always occur at. As more genes are deleted, the local strength of the fibre increases, but the final failure force depends on the weakest septum or flaw. Thus, deleting more genes does not increase the tensile strength of the fibre as expected. Instead, reinforcing the septa is extremely important for improving the mechanical properties of multiple-gene-deleted fibres.

### Variability in the mechanical properties of *B. subtilis* multicellular fibres and use of Weibull analysis

The experimental data obtained from tensile tests reveal that the multicellular fibres have highly variable mechanical properties (Fig. 1). Generally, in addition to the differences between individual organisms, any changes in loading rate, sample moisture content, and biological pre-treatments can change the mechanical properties. In particular, *B. subtilis* multicellular fibres retain septa, which are the weak links of fibres, with various separation levels. Therefore, the more septa in a fibre, the more their mechanical properties will vary.

Considering the variation of mechanical properties and the effect of length on the tensile strength obtained from the tensile tests, we introduce the modified Weibull distribution^40^ based on the weakest-link concept to explain the variation mechanism. In addition to the existence of internal flaws and morphological or geometrical changes, if we assume that the tensile strengths of the septa in a fibre are random and independent, then the Weibull distribution can predict the variability in the fibres’ tensile strength and the length effect.

Based on the weakest-link theory, Wagner *et al*. proposed the modified Weibull model^40^,^41^:


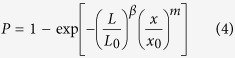


where *P* is the failure probability of a long fibre connected by *n* independent segments, *x* is the tensile strength, *L* is the gauge length, *L*_0_ is the length of a unit link of the fibre, *m* is the Weibull modulus (shape parameter), *β* is a parameter (0 < *β* < 1), and *x*_0_ is a scale parameter.

Letting *y* = *xL*^*β/m*^ and *y*_0_ = *x*_0_*L*_0_^*β/m*^, equation (4) becomes


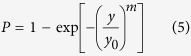


Taking the natural logarithm of equation (5) gives


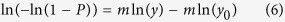


From equation (6), ln(*y*) is linearly proportional to ln(−ln(1−*P*)), and the slope is the Weibull modulus *m*, which indicates the dispersion in fibre strength. The lower the *m*, the higher the variability. Therefore, if we experimentally obtain values of ln(*y*) and ln(−ln(1−*P*)), then *m* and *y*_0_ can be determined. The average tensile strength 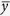 and its standard deviation σ (or coefficient of variation (CV)) can then be calculated by ref. 42


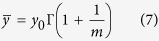



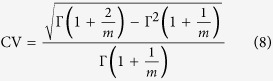


**Figure 6:**
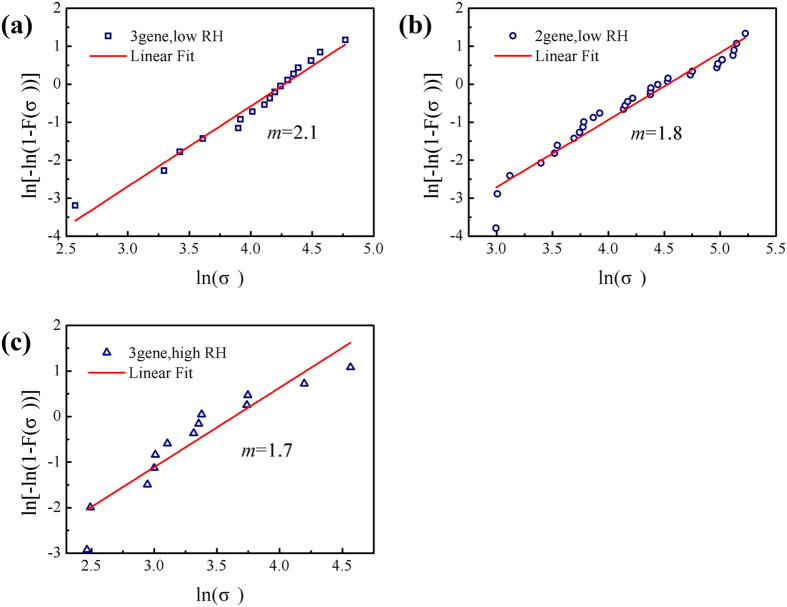
Modified Weibull distribution of the fibres’ average tensile strength. (**a**,**b**) Tensile strength at low RH. Weibull distribution parameters can be obtained by fitting the experimental data to the linear function in equation (6) using the least-squares method. For multicellular fibres with three genes deleted, their Weibull modulus *m* and average tensile strength 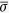 are 2.1 and 63.3 MPa, respectively; for multicellular fibres with two genes deleted, they are 1.8 and 82.5 MPa, respectively. (**c**) Tensile strength at high RH. The parameters *m* and 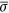 are 1.7 and 33.7 MPa, respectively, for fibres with three genes deleted.

Figure 6 shows typical Weibull plots of ln(−ln(1−*P*)) versus ln(*y*) for the tensile strength of fibres with three and two genes deleted at low and high RH, respectively. The good linearity of the curves and the low Weibull modulus show that the individual fibres have highly variable tensile strength. The sources of variability in the tensile strength are the weak points (e.g., internal flaws), geometrical changes, and weak-link septa within the gauge length of the fibres. By fitting the curves and combining equations (7) and (8), it is found that the tensile strength of multicellular fibres obey Weibull statistics. The Weibull average tensile strength (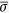), standard deviation (CV), and Weibull modulus (*m*) are 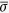 = 63.3 MPa, CV = 0.5 and *m* = 2.1 for fibres with three genes deleted, and 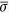 = 82.5 MPa, CV = 0.6 and *m* = 1.8 for fibres with two genes deleted at low RH. The fibres with three genes deleted at high RH have 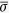 = 33.7 MPa, CV = 0.6 and *m* = 1.7.

These results indicate that fibres at various RH levels have similar variations in tensile strength, and that the flaws and weak-link septa still dominate their variability in tensile strength. Further, their low Weibull modulus indicates a large dispersity of tensile strength. Examples in the literature include *m* = 1.7 for multi-walled carbon nanotubes (MWCNTs) with a 10 ± 4 μm gauge length^43^, *m* = 4.5 for Thornel-300 (T300) carbon fibres with a 60 mm gauge length^44^, and *m* = 5.1 for glass fibres with a 5 mm gauge length^45^. Comparing these typical materials with the multicellular fibres in this study, the tensile strengths of the multicellular fibres and MWCNTs have a greater dispersion owing to their smaller gauge lengths, while the T300 carbon fibres and glass fibres show consistent measured tensile strengths owing to their much larger gauge lengths.

Therefore, although *B. subtilis* multicellular fibres have a high elastic modulus, similar to those of other natural fibres (Table 1), the large dispersion of their tensile strengths may limit the engineering applicability of single ones. The mechanical properties of these multicellular fibres can be improved by eliminating the septa among pairs of cells, increasing the cross-linking in the cell wall through chemical methods, or/and increasing the amount of peptidoglycan in the cell wall.

## Conclusions

For the first time, we directly stretched individual *B. subtilis* multicellular fibres with a diameter of about 0.7 μm and a length in the range of 25.7–254.3 μm by site-specific mutation and measured their mechanical properties using a testing system developed here. The elastic modulus of the fibres is on the same order of magnitude as those of natural fibres such as coir, but their tensile strength is much lower. The fibre length affects their tensile properties, presumably because of the presence of septa. Humidity also strongly affects the deformation behaviour of the fibres, which could be attributed to many hydrogen bonds among the peptides in peptidoglycan. Two theoretical models and numerical calculations are employed to characterise the tensile behaviour of the fibre at low and high RH, respectively. Loading and unloading experiments demonstrate that the periodic necking instability comes from structural changes (septa) and viscoelasticity dominates deformation of the multicellular fibres at high RH. We expect that these findings on individual multicellular fibres to provide the mechanisms through which their mechanical performance can be improved for engineering applications.

## Methods

### Material and sample preparation

#### Strains and culture conditions

Mutants of *B. subtilis* strain 168 were used to generate multicellular fibres, and *E. coli* strains were used to construct plasmids. Table 3 lists the used genotypes and derivations of the *B. subtilis* strains. All of the bacteria were cultivated in Luria–Bertani (LB) broth containing 10 g/L tryptone, 5 g/L yeast extract, and 10 g/L NaCl at 37 °C under 200 rpm rotary shaking, or on LB agar plates containing 1.5% Sigma agar at 37 °C. Whenever appropriate, antibiotics were added to the medium in the following concentrations: 5 μg/mL chloramphenicol or 100 μg/mL ampicillin.

#### Plasmid construction

pCU was used as a shuttle vector for plasmid construction in *E. coli* and for gene deletion in *B. subtilis*. The pCU plasmid has a replicon and an ampicillin resistance gene (*bla*) in *E. coli* and a chloramphenicol resistance gene (*cat*) in *B. subtilis.* However, it has no replication origin in *B. subtilis*, so it must be integrated into the *B. subtilis* genome via homologous recombination to express the chloramphenicol resistance gene. The *sigD, lytE*, and *lytD* genes were deleted, and the corresponding plasmids used for deletion are called pCU-sigD, pCU-lytE, and pCU-lytD, respectively. To remove *sigD*, the pCU-sigD plasmid was constructed: one kilo base pair sequence upstream *sigD* and one kilo base pair sequence downstream *sigD* on the genome were amplified by polymerase chain reaction (PCR), and 5 μg plasmid pCU was digested by the fast digestion restriction enzymes XbaI and BamHI (Fermentas) to transform the circular plasmid into a linear one. Then the two PCR-amplified fragments were ligated in tandem into a linear plasmid using Gibson assembly^46^. The colonies were spread on the ampicillin agar plates for selection, and positive colonies after sequencing were used for transformation. All of the plasmids used for gene deletion were constructed using the same method.

#### Plasmid transformation into B. subtilis

This transformation was performed based on a previously described method^47^. First, the *B. subtilis* 168 Δ*upp* strain was inoculated into GM1 medium (5 mL; 0.8% glucose, 0.04% acid-hydrolysed casein, 0.1% yeast extract, 0.02% MgSO_4_, Spizizen’s salt, 50 μg/mL tryptophan) and cultured overnight for 8–20 h. The Spizizen’s salt comprised 2% (NH_4_)_2_SO_4_, 6% KH_2_PO_4_, 18.3% K_2_HPO_4_, and 1.2% sodium citrate. A sample of the culture (750 μL) was then inoculated into a fresh GM2 medium (5 mL; 0.8% glucose, 0.02% acid-hydrolysed casein, 0.16% MgSO_4_, Spizizen’s salt, and 50 μg/mL tryptophan) and cultured for 1.5 h. A sample of this culture (500–1000 μL) was transferred into a sterile 1.5-mL tube, mixed with 0.1–10.0 μg plasmid, which were incubated at 37 °C with shaking at 200 rpm for another 1 h. Finally, the bacterial mixture was spread onto the LB agar medium with chloramphenicol antibiotic and incubated at 37 °C overnight.

#### Mutant construction

Mutants were constructed using a previously described method^48^. The colonies on chloramphenicol-containing LB agar were inoculated into another fresh LB medium and cultured for 6–12 h. A sample of the culture (200 μL) was then spread onto a 5-F-U plate, which was composed of 0.4% glucose, 0.2% glutamine, 5 μM MnSO_4_, 1 μg/mL VB1, 100 μg/mL amino acid mix^49^, 25 μM 5-fluorouracil, mineral mix (5.4% K_2_HPO_4_, 3% KH_2_PO_4_, 0.5% sodium citrate, 0.1% MgSO_4_ and 1% K_2_SO_4_), and 1.7% agar. After cultivation for 12–36 h at 37 °C, colonies on the plates were selected and verified by polymerase chain reaction. Normally, only half of the colonies were positive. After deleting one gene, other genes were deleted one-by-one to construct the multiple-gene mutants.

### Testing system and measurement method

#### Tested B. subtilis multicellular fibres

**Figure 7:**
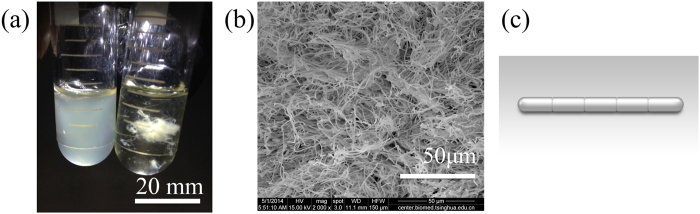
Materials. (**a**) Photograph of *B. subtilis* 168 (left) and *B. subtilis* multicellular fibres (right). (**b**) SEM image of the disorderly-stacked fibres. (**c**) An individual chain-like fibre composed of bacterial cells connected by septa.

Figure 7a shows *B. subtilis* 168 and *B. subtilis* multicellular fibres in tests, and Fig. 7b also shows the disorderly-stacked *B. subtilis* multicellular fibres observed under the SEM. An individual fibre is a chain of cells (Fig. 7c) connected by septa at different separation levels. The length of a *B. subtilis* 168 is 4 μm, while the length of the longest fibre reaches a few millimetres, and their diameters vary from 0.5 to 0.9 μm. The fibres were freeze-dried, stored in a sealed container at room temperature and tested within 1 week of formation.

#### Testing system

**Figure 8:**
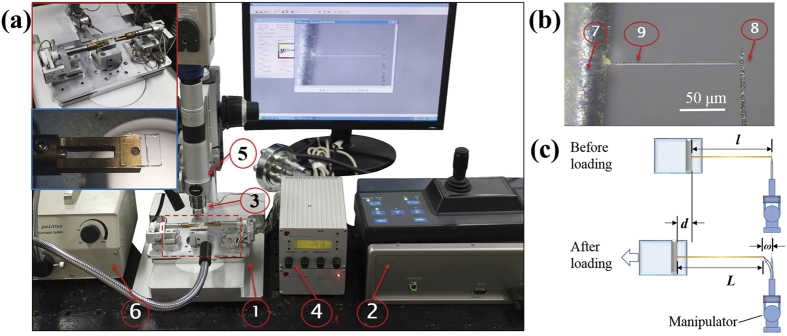
Testing platform and measurement method. (**a**)Tensile testing system. ①m-MTS system. ②m-MTS control unit. ③Micro/nanomanipulator. ④Micro/nanomanipulator control unit. ⑤ Optical microscope. ⑥ Microscope light source. The inset shows an enlarged view of the selected area. (**b**) A reliable structure for uniaxial tensile testing of an individual fibre is formed. ⑦ A 100-μm-diameter tungsten wire fixed on the 5 × 5 mm^2^ transparent plate. ⑧ Force-sensing probe. ⑨ Individual *B. subtilis* multicellular fibre. (**c**) Schematic of the tensile testing.

To measure the mechanical properties of the fibres, we built a precise tensile platform (see the enlarged view of the selected area in Fig. 8a) with a two-level design to realize both coarse and fine displacement loading. The main components of the tensile platform were two sets of three-axis (*xyz*-axis) coarse translation stages and two sets of three-axis (*xyz*-axis) fine translation stages, which allowed us to use displacement loading from millimetres to nanometres. The coarse translation stages used piezoelectric motors with a travel distance of 12 mm and a displacement resolution of 0.2 μm, and the fine translation stages used piezoelectric stacks with a travel distance of 6 μm and a displacement resolution of 0.5 nm. To realize the two-level force measurement, two types of exchangeable force sensors were assembled. For the coarse level, a strain gage force sensor was made with a force range of 200 mN and a resolution of 0.1 mN. The linearity and repeatability is 0.1% of the full scale. Also, the average stiffness coefficient is 1.25 mN/μm. For the fine level, a commercially capacitive microforce sensing probe (FT-S100, FemtoTools, Switzerland) is used with a force range of 100 μN and a resolution of 5 nN. In addition to these two microforce sensors, calibrated cantilever-type force sensors are also used in the practical measurements to adapt to the tested samples. This platform, which we call the micro/nanoscale material testing system (m-MTS), can perform mechanical tests of tension, compression, and bending under an OM (HIScope KH-3000, Hirox, Tokyo, Japan) or SEM (Quanta 450 FEG, FEI, USA). We verified the repeatability of the m-MTS by *in situ* tensile testing of carbon fibres (T300B-6000, TORAY, Japan) as a reference material. Table 4 lists the tensile properties measured from five tests of fibres (average gauge length of 500 μm and diameter of 7 μm). The measured properties of these fibres are tensile modulus of 216 ± 16 GPa, tensile strength of 3.2 ± 0.5 GPa, and elongation of 1.4 ± 0.2%, which agree with reference values^50^. The good repeatability of these results also demonstrate the performance of the m-MTS.

To manipulate and clamp the multicellular fibres for the tensile tests, a micro/nanomanipulator (MM3A-EM, Kleindiek Nanotechnik GmbH, Reutlingen, Germany) with a cantilever tungsten probe (made by electrochemical etching) was attached to the m-MTS (see the enlarged view of the selected area in Fig. 8a). The micro/nanomanipulator had three degrees of freedom operating in modes of left/right, up/down, and in/out with displacement resolutions of 5, 3.5, and 0.5 nm, respectively. In testing the multicellular fibres, the m-MTS was used as the loading platform, while the calibrated cantilever tungsten probe was used as the force sensor^51^. Figure 8a shows the whole system used to test the multicellular fibres.

#### Liquid drop method

To test the tensile properties of individual fibres on the OM stage, we prepared a special clamping end consisting of a 5 × 5 mm^2^ transparent plate (cut from a coverslip) with a 100-μm-diameter tungsten wire attached to its surface, and mounted it to the left arm of the m-MTS (see the enlarged view of the selected area in Fig. 8a). The transparent clamping plate allows for transmission illumination of the fibre, producing a clear microscopic view during the experiment. The liquid drop method (LDM) is proposed to separate the individual fibre from clusters and form a reliable structure for uniaxial stretching, as shown in Fig. 8b.

**Figure 9:**
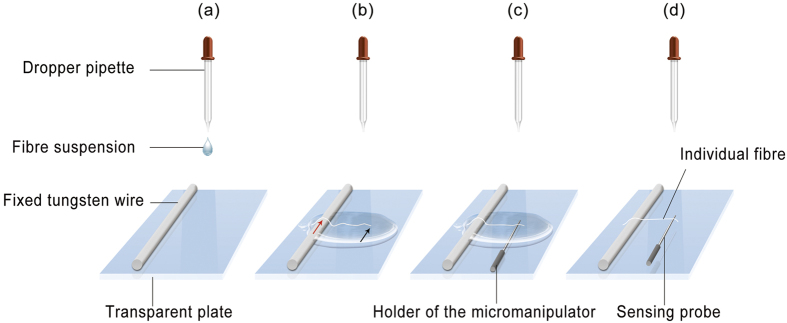
Procedure of the liquid drop method (LDM). (**a**) A drop of fibre suspension is transferred to the surface of the transparent plate to cover the fixed tungsten wire. (**b**) One end of a fibre is adhered to the tungsten wire by capillary force (denoted by the red arrow), while the other end is free (denoted by the black arrow). (**c**) A sensing probe controlled by the micro/nanomanipulator moves to the free end of the selected fibre and clamps it with the aid of the capillary force. (**d**) A reliable uniaxial tension structure of the individual fibre is formed.

**Figure 10:**
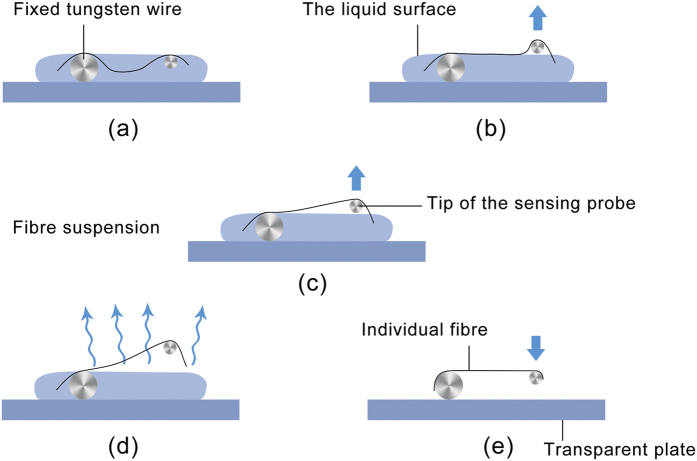
Process of manipulating the probe. (**a**)The sensing probe is moved to the free end of a fibre. (**b**,**c**) The probe is lifted until the tip is detached from the liquid surface. (**d**) The whole system is kept stationary until the suspension evaporates. (**e**) The probe is lowered to recover the fibre in the horizontal plane.

The LDM is performed as follows (Fig. 9). First, a small amount of freeze-dried multicellular fibres are added to deionized water to obtain an appropriate concentration. Then a tiny drop of the fibre suspension is transferred by a dropper pipette to the transparent clamping plate, covering the fixed 100-μm-diameter tungsten wire (Fig. 9a) which raises the liquid surface by 100 μm (Fig. 10a). Because fibres are dispersed in the dropped suspension, some of them will adhere to the fixed tungsten wire by the capillary force at one end (red arrow in Fig. 9b), while the other end of the fibres are free (black arrow in Fig. 9b).

The sensing tungsten probe controlled by the micro/nanomanipulator is moved to the free end of a fibre (Figs 9c and 10a), and the probe is lifted by the up/down mode of the micro/nanomanipulator, until the tip is detached from the liquid surface (Fig. 10b and c). In this process, the probe tip overcomes the surface tension and breaks the liquid surface, causing the free end of the fibre to be clamped with the aid of the capillary force.

At this point, the whole system is kept static until the suspension evaporates, exposing the entire length of the fibre to air (Fig. 10d). To achieve collimation stretching, fibre alignment could be accomplished by adjusting the micro/nanomanipulator or the m-MTS during evaporation. After alignment completion and an additional waiting period of 20 min, the fibre will be dried owing to gradual evaporation of the suspension. Both ends are strongly adhered to the fixed 100-μm-diameter tungsten wire and probe tip by the surface adhesion effect and van der Waals interaction. Then the fibre is recovered in the horizontal plane by the up/down mode of the micro/nanomanipulator (Fig. 10e), forming a reliable structure for uniaxial tensile testing of the individual fibre (Fig. 9d). Note that the water is just used as a medium to help transfer the fibre. All of the tensile tests are conducted after the water evaporates, avoiding all effects from a liquid environment. In fact, slip failure rarely appears at the clamping ends during testing, which suggests that this clamping method is highly effective.

The LDM is a simple and effective method for producing a reliable uniaxial tension/compression structure with the help of an OM and manipulators, avoiding any complicated processes in picking up, transferring, and clamping. This method is suitable for moisture-sensitive low-dimensional materials (e.g. one-dimensional materials with length from tens of microns to hundreds of microns and diameters at the micron and submicron scales), especially for materials which are disorderly-stacked, and materials that are brittle when dry yet flexible when in a liquid environment. Also, the lack of slip failure at the clamping ends during the test suggests that the clamping strength is at least 12 μN. Because this method requires a liquid medium at first, it cannot be directly applied under vacuum.

#### Tensile force and displacement measurements

During tensile testing, the micro/nanomanipulator is held static and the fibre is stretched by the displacement-control mode of the m-MTS which controls the motion of the fixed 100-μm-diameter tungsten wire. Note that the wire is sufficiently stiff to serve as non-deformable clamping end. The whole tension process is recorded by a charge-coupled device (CCD) connected to the OM. The elongation of the fibre and the deflection of the tip of the force-sensing probe are obtained by analysing the image sequence. The load of the fibre is calculated from the deflection of the tip based on beam theory; considering that the root of probe is fixed on the micro/nanomanipulator, the deflection of the tip is entirely caused by the tension of the fibre. Figure 8c shows a schematic of the stretching process. By loading the fixed 100-μm-diameter tungsten wire, the fibre is stretched; the elongation of the fibre is *L*−*l* and the load is *kw*, where *l* is the initial length of the fibre, *L* is the current length of the fibre, *k* is the spring constant of the probe, and *w* is the deflection of the probe tip. This method can obtain the elongation and load of the fibre at every moment.

## Additional Information

**How to cite this article**: Ye, X. *et al*. Study of the tensile properties of individual multicellular fibres generated by *Bacillus subtilis. Sci. Rep.* **7**, 46052; doi: 10.1038/srep46052 (2017).

**Publisher's note:** Springer Nature remains neutral with regard to jurisdictional claims in published maps and institutional affiliations.

## Supplementary Material

Supplementary Information

## Figures and Tables

**Table 1:** Mechanical properties of multicellular fibres with various gene deletions and other natural fibres.

Material	Diameter (μm)	Tensile strength (MPa)	Elastic modulus (GPa)	Breaking strain (%)	Source
Δ*SED*	0.7	62.2	5.8	1.5	This study
Δ*SE*	0.7	82.7	4.5	2.4	This study
*B. mori* silk	9.3	650	16	7–16	[27]
Coir	100–450	153	4–6	15–40	[28, 29]
Jute	25–120	533	2.5–13.0	1–2	[28, 29]
Cotton	11–22	287–597	5.5–12.6	7.0–8.0	[28]

Using the targeted gene deletion method, two types of *B. subtilis* multicellular fibres are synthesized: Δ*SED* (fibres with *sigD, lytE*, and *lytD* genes deleted) and Δ*SE* (fibres with *sigD* and *lytE* genes deleted). Their mechanical properties are compared with those of *Bombyx mori (B. mori*) silk, coir, jute, and cotton at low relative humidity (RH).

**Table 2:** Elastic modulus and viscosity of some multicellular fibres at high RH (46–56%).

Sample	RH (%)	Length (μm)	Elastic modulus (MPa)	Viscosity (MPa s)
1	46	146.7	71.0	215.2
2	46	119.0	80.0	63.9
3	51	117.0	47.3	49.9
4	52	100.4	63.7	136.0
5	56	129.0	59.9	83.8

For Δ*SED* fibres (fibres with *sigD, lytE*, and *lytD* genes deleted), the mechanical parameters are obtained by fitting the experimental results at high RH with the Kelvin–Voigt model.

**Table 3:** Strains and plasmids used in the present study.

Strains or Plasmids	Genotypes	Derivations
Strains		
BS168	*Bacillus subtilis TrpC2*	From TTCTT
BS168 ΔUpp	*Bacillus subtili TrpC2 Δupp*	Donated by Tianjin University/China
ΔS	*TrpC2ΔuppΔsigD*	pCU-sigD → BS168 ΔUpp
ΔE	*TrpC2ΔuppΔlytE*	pCU-lytE → BS168 ΔUpp
ΔD	*TrpC2Δupp ΔlytD*	pCU-lytD → BS168 ΔUpp
ΔSED	*TrpC2ΔuppΔsigDΔlytEΔlytD*	pCU-lytD → ΔSE
Plasmids		
pCU	*bla cat*	Donated by Tianjin University/China
pCU-sigD	*bla cat ΔsigD*	This study
pCU-lytE	*bla cat ΔlytE*	This study
pCU-lytD	*bla cat ΔlytD*	This study

**Table 4:** Tensile properties of carbon fibres.

Sample	Tensile modulus (GPa)	Tensile strength (GPa)	Breaking strain (%)
1	218	2.6	1.2
2	203	3.5	1.5
3	208	2.5	1.2
4	245	3.9	1.6
5	204	3.5	1.7

## References

[b1] Kumar V., Bhardwaj Y. K., Rawat K. P. & Sabharwal S. Radiation-induced grafting of vinylbenzyltrimethylammonium chloride (VBT) onto cotton fabric and study of its anti-bacterial activities. Radiat. Phys. Chem. 73, 175–182 (2005).

[b2] Hakimi O., Knight D. P., Vollrath F. & Vadgama P. Spider and mulberry silkworm silks as compatible biomaterials. Compos. Pt. B: Eng. 38, 324–337 (2007).

[b3] Schleifer K. H. & Kandler O. Peptidoglycan types of bacterial cell walls and their taxonomic implications. Bacteriol. Rev. 36, 407–477 (1972). 4568761 10.1128/br.36.4.407-477.1972 PMC408328

[b4] Uehara T., Dinh T. & Bernhardt T. G. LytM-domain factors are required for daughter cell separation and rapid ampicillin-induced lysis in *Escherichia coli*. J. Bacteriol. 191, 5094–5107 (2009). 19525345 10.1128/JB.00505-09 PMC2725582

[b5] Typas A., Banzhaf M., Gross C. A. & Vollmer W. From the regulation of peptidoglycan synthesis to bacterial growth and morphology. Nature Rev. Microbiol. 10, 123–136 (2012). 10.1038/nrmicro2677 PMC5433867 22203377

[b6] Saxe C. L. & Mendelson N. H. Morphological and genetic characterization of a bacteriophage-resistant *Bacillus subtilis* macrofiber-producing strain. J. Bacteriol. 157, 109–114 (1984). 6418716 10.1128/jb.157.1.109-114.1984 PMC215137

[b7] Chen R., Guttenplan S. B., Blair K. M. & Kearns D. B. Role of the σ^D^-dependent autolysins in *Bacillus subtilis* population heterogeneity. J. Bacteriol. 191, 5775–5784 (2009). 19542270 10.1128/JB.00521-09 PMC2737971

[b8] Davis S. A. . Brittle bacteria: A biomimetic approach to the formation of fibrous composite materials. Chem. Mater. 10, 2516–2524 (1998).

[b9] Thwaites J. J. & Mendelson N. H. Biomechanics of bacterial walls: studies of bacterial thread made from *Bacillus subtilis*. Proc. Natl Acad. Sci. USA 82, 2163–2167 (1985). 3920662 10.1073/pnas.82.7.2163 PMC397513

[b10] Lan G., Wolgemuth C. W. & Sun S. X. Z-ring force and cell shape during division in rod-like bacteria. Proc. Natl Acad. Sci. USA 104, 16110–16115 (2007). 17913889 10.1073/pnas.0702925104 PMC2042170

[b11] Vollmer W. & Seligman S. J. Architecture of peptidoglycan: more data and more models. Trends Microbiol. 18, 59–66 (2010). 20060721 10.1016/j.tim.2009.12.004

[b12] Touhami A., Nysten B. & Dufrêne Y. F. Nanoscale mapping of the elasticity of microbial cells by atomic force microscopy. Langmuir 19, 4539–4543 (2003).

[b13] Hoh J. H. & Schoenenberger C. Surface morphology and mechanical properties of MDCK monolayers by atomic force microscopy. J. Cell Sci. 107, 1105–1114 (1994). 7929621 10.1242/jcs.107.5.1105

[b14] Zhao L. M., Schaefer D. & Marten, M. R. Assessment of elasticity and topography of *Aspergillus nidulans* spores via atomic force microscopy. Appl. Environ. Microbiol. 71, 955–960 (2005). 15691953 10.1128/AEM.71.2.955-960.2005 PMC546822

[b15] Vadillo-Rodriguez V., Beveridge T. J. & Dutcher J. R. Surface viscoelasticity of individual gram-negative bacterial cells measured using atomic force microscopy. J. Bacteriol. 190, 4225–4232 (2008). 18408030 10.1128/JB.00132-08 PMC2446760

[b16] Vadillo-Rodriguez V., Schooling S. R. & Dutcher J. R. *In situ* characterization of differences in the viscoelastic response of individual gram-negative and gram-positive bacterial cells. J. Bacteriol. 191, 5518–5525 (2009). 19581369 10.1128/JB.00528-09 PMC2725611

[b17] Vadillo-Rodríguez V. & Dutcher J. R. Viscoelasticity of the bacterial cell envelope. Soft. Mat. 7, 4101–4110 (2011).

[b18] Tuson H. H. . Measuring the stiffness of bacterial cells from growth rates in hydrogels of tunable elasticity. Mol. Microbiol. 84, 874–891 (2012). 22548341 10.1111/j.1365-2958.2012.08063.x PMC3359400

[b19] Amir A., Babaeipour F., McIntosh D. B., Nelson D. R. & Jun S. Bending forces plastically deform growing bacterial cell walls. Proc. Natl Acad. Sci. USA 111, 5778–5783 (2014). 24711421 10.1073/pnas.1317497111 PMC4000856

[b20] Mendelson N. H. & Thwaites J. J. Cell wall mechanical properties as measured with bacterial thread made from *Bacillus subtilis*. J. Bacteriol. 171, 1055–1062 (1989). 2492505 10.1128/jb.171.2.1055-1062.1989 PMC209701

[b21] Thwaites J. J. & Mendelson N. H. Mechanical properties of peptidoglycan as determined from bacterial thread. Int. J. Biol. Macromol. 11, 201–206 (1989). 2518734 10.1016/0141-8130(89)90069-x

[b22] Thwaites J. J. & Surana U. C. Mechanical properties of *Bacillus subtilis* cell walls: effects of removing residual culture medium. J. Bacteriol. 173, 197–203 (1991). 1898920 10.1128/jb.173.1.197-203.1991 PMC207175

[b23] Thwaites J. J., Surana U. C. & Jones A. M. Mechanical properties of *Bacillus subtilis* cell walls: effects of ions and lysozyme. J. Bacteriol. 173, 204–210 (1991). 1898921 10.1128/jb.173.1.204-210.1991 PMC207176

[b24] Margot P., Wahlen M., Gholamhuseinian A., Piggot P. & Karamata D. The lytE gene of *Bacillus subtilis* 168 encodes a cell wall hydrolase. J. Bacteriol. 180, 749–752 (1998). 9457885 10.1128/jb.180.3.749-752.1998 PMC106949

[b25] Lazarevic V., Margot P., Soldo B. & Karamata D. Sequencing and analysis of the *Bacillus subtilis* lytRABC divergon: a regulatory unit encompassing the structural genes of the N-acetylmuramoyl-L-alanine amidase and its modifier. Microbiol. 138, 1949–1961 (1992). 10.1099/00221287-138-9-1949 1357079

[b26] Rogers H. J., Perkins H. R. & Ward J. B. Microbial cell walls and membranes. (London, UK: Chapman and Hall, 1980).

[b27] Perez-Rigueiro J., Viney C., Llorca J. & Elices M. Mechanical properties of single-brin silkworm silk. J. Appl. Polym. Sci. 75, 1270–1277 (2000).

[b28] Bledzki A. K. & Gassan J. Composites reinforced with cellulose based fibres. Prog. Polym. Sci. 24, 221–274 (1999).

[b29] Bisanda E. & Ansell M. P. Properties of sisal-CNSL composites. J. Mater. Sci. 27, 1690–1700 (1992).

[b30] Turner R. D., Vollmer W. & Foster S. J. Different walls for rods and balls: The diversity of peptidoglycan. Mol. Microbiol. 91, 862–874 (2014). 24405365 10.1111/mmi.12513 PMC4015370

[b31] Hayhurst E. J., Kailas L., Hobbs J. K. & Foster S. J. Cell wall peptidoglycan architecture in *Bacillus subtilis*. Proc. Natl Acad. Sci. USA 105, 14603–14608 (2008). 18784364 10.1073/pnas.0804138105 PMC2567149

[b32] Egan A. J. F. & Vollmer W. The physiology of bacterial cell division. Ann. NY Acad. Sci. 1277, 8–28 (2013). 23215820 10.1111/j.1749-6632.2012.06818.x

[b33] Adams D. W. & Errington J. Bacterial cell division: assembly, maintenance and disassembly of the Z ring. Nature Rev. Microbiol. 7, 642–653 (2009). 19680248 10.1038/nrmicro2198

[b34] Plaza G. R. . Effect of water on Bombyx mori regenerated silk fibers and its application in modifying their mechanical properties. J. Appl. Polym. Sci. 109, 1793–1801 (2008).

[b35] Perez-Rigueiro J., Viney C., Llorca J. & Elices M. Mechanical properties of silkworm silk in liquid media. Polymer 41, 8433–8439 (2000).

[b36] Zhang B., Davis S. A., Mendelson N. H. & Mann S. Bacterial templating of zeolite fibres with hierarchical structure. Chem. Commun. 9, 781–782 (2000).

[b37] Field M. . Ordering nanometer-scale magnets using bacterial thread templates. Appl. Phys. Lett. 73, 1739–1741 (1998).

[b38] Davis S. A., Breulmann M., Rhodes K. H., Zhang B. & Mann S. Template-directed assembly using nanoparticle building blocks: a nanotectonic approach to organized materials. Chem. Mater. 13, 3218–3226 (2001).

[b39] Davis S. A., Burkett S. L., Mendelson N. H. & Mann S. Bacterial templating of ordered macrostructures in silica and silica-surfactant mesophases. Nature 385, 420–423 (1997).

[b40] Cohen A. C. Maximum likelihood estimation in the Weibull distribution based on complete and on censored samples. Technometrics 7, 579–588 (1965).

[b41] Friedrich K. Application of fracture mechanics to composite materials. (New York, USA, 2012).

[b42] Zhang Y., Wang X., Pan N. & Postle R. Weibull analysis of the tensile behavior of fibers with geometrical irregularities. J. Mater. Sci. 37, 1401–1406 (2002).

[b43] Barber A. H., Andrews R., Schadler L. S. & Wagner H. D. On the tensile strength distribution of multiwalled carbon nanotubes. Appl. Phys. Lett. 87, 203106 (2005).

[b44] Chi Z., Chou T. W. & Shen G. Determination of Single fibre strength distribution from fibre bundle testings. J. Mater. Sci. 19, 3319–3324 (1984).

[b45] Andersons J., Joffe R., Hojo M. & Ochiai S. Glass fibre strength distribution determined by common experimental methods. Compos. Sci. Technol. 62, 131–145 (2002).

[b46] Gibson D. G. . Enzymatic assembly of DNA molecules up to several hundred kilobases. Nat. Methods 6, 343–345 (2009). 19363495 10.1038/nmeth.1318

[b47] Anagnostopoulos C. & Spizizen J. Requirements for transformation in *Bacillus subtilis*. J. Bacteriol. 81, 741–746 (1961). 16561900 10.1128/jb.81.5.741-746.1961 PMC279084

[b48] Fabret C., Ehrlich S. D. & Noirot P. A new mutation delivery system for genome-scale approaches in *Bacillus subtilis*. Mol. Microbiol. 46, 25–36 (2002). 12366828 10.1046/j.1365-2958.2002.03140.x

[b49] Saxild H. H. & Nygaard P. Genetic and physiological characterization of *Bacillus subtilis* mutants resistant to purine analogs. J. Bacteriol. 169, 2977–2983 (1987). 3110131 10.1128/jb.169.7.2977-2983.1987 PMC212336

[b50] Physical property table of Torayca yarn. Available at: http://www.torayca.com/en/download/pdf/torayca.pdf (2016) (Date of access: 21 November 2016).

[b51] Li X., Su D. & Zhang Z. A novel technique of microforce sensing and loading. Sens. Actuators, A. 153, 13–23 (2009).

