# Supplementary material for: Study of the tensile properties of individual multicellular fibres generated by Bacillus subtilis
Source: Sci Rep. 2017 Apr 5;7:46052. doi: 10.1038/srep46052 (PMC5380956; doi:10.1038/srep46052)
Supplement: Supplementary Information [file srep46052-s1.pdf]

Study of the tensile properties of individual multicellular fibres generated by *Bacillus subtilis*

Xuan Ye, Liang Zhao, Jiecun Liang, Xide Li*& Guo-Qiang Chen*

Loading and unloading experiment.

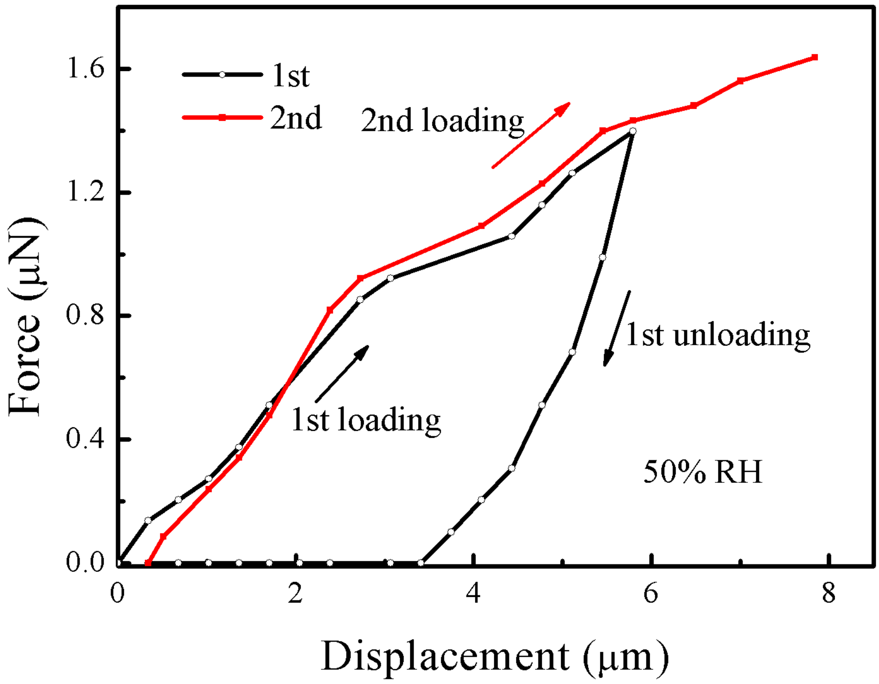

Figure S1. The results of loading and unloading. The fibre recovers to its initial length after unloading, and then, a hysteresis loop is also formed.

Finite element analysis of the tension of multicellular fibres.

Because the fibres show a continuous cylindrical wall with invagination at the positions of septa at varying separation levels (Fig. S2a), previous theoretical models (i.e., the standard solid model and Kelvin–Voigt model) cannot fully characterise how the septum affects the deformation. Therefore, we carry out the finite element analysis in detail.

An individual *B. subtilis* multicellular fibre contains several to hundreds of cells subjected to suppressive separation. In other words, there are n ($n \geq 2$) cells in series

with $n-1$ septa with varying separation levels during growth. Considering the cell division process[1,2] and typical transmission electron microscopy (TEM) images (Fig. S2a), we construct mechanical models of multicellular fibres with septa at different separation levels to study how their morphology changes under uniaxial tension. The models of the positions of septa (Fig. S2b) and fibres (Fig. S3) at three separation levels (early, middle and late stages in cell separation) are shown. Actually, an individual fibre is connected by septa with various separation levels, so a fibrous model contains several septa at two separation levels (early and late stages in cell separation) as an example is constructed in Fig. S4.

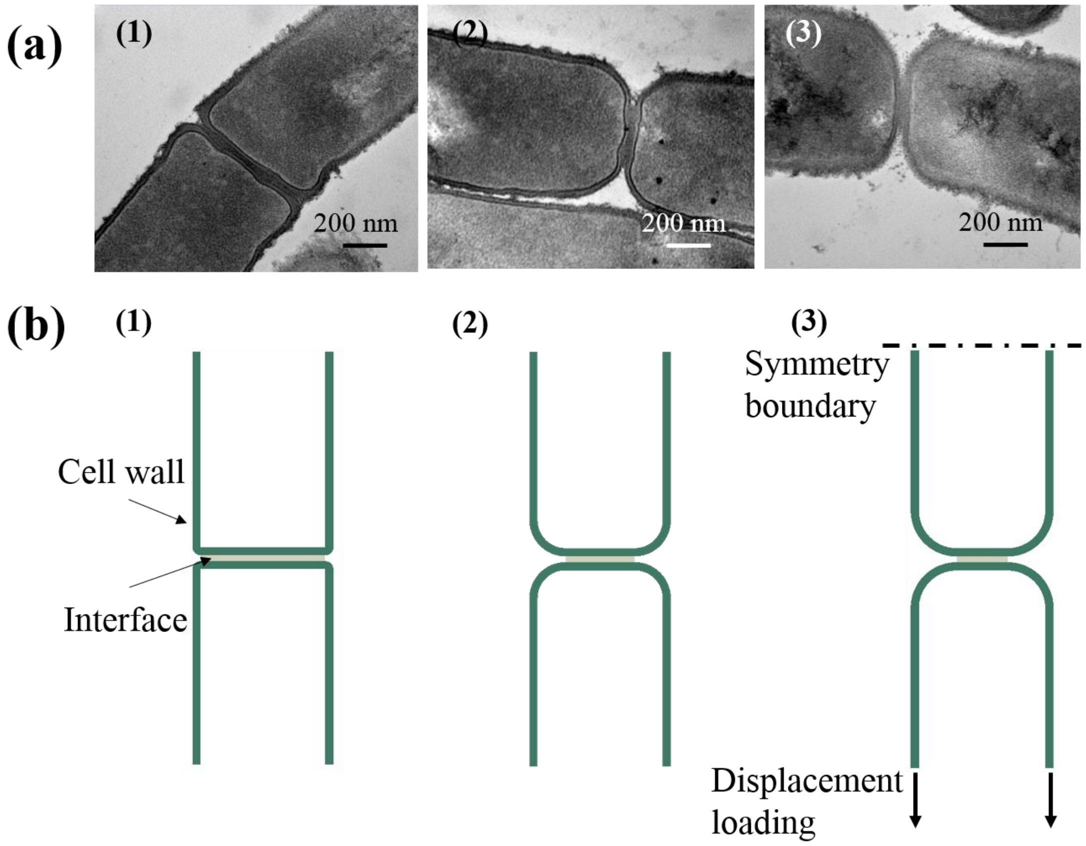

Figure S2. (a) Transmission electron micrographs of *B. subtilis* cells in longitudinal section at three separation levels (early, middle and late stages in cell separation). **(b)** Finite element analysis models corresponding to Fig. S2a (1), 2a (2) and 2a (3).

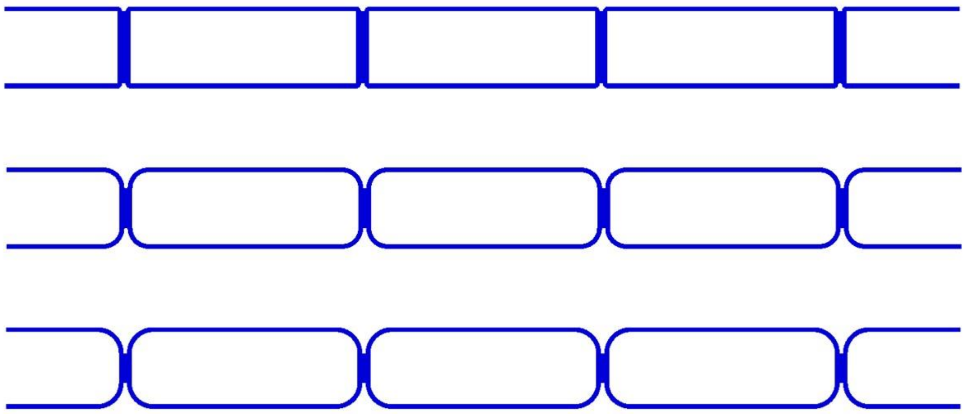

Figure S3. Fibrous models for three separation levels (corresponding to Fig. S2b (1), 2b (2) and 2b (3)).

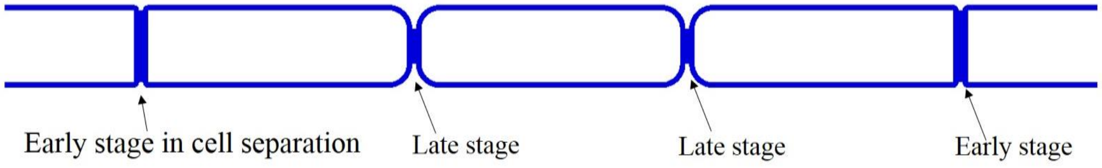

Figure S4. An example of a fibrous model contains several septa at different separation levels.

We studied their deformation behaviour by using viscoelastic constitutive relation (Poynting–Thomson model[3]). The material parameters of cell wall are obtained by fitting the experimental curve (E_1 = 500 MPa, E_2 = 100 MPa, and η_1 = 127 MPa s). The intermediate interface layer is chosen to be softer than the cell wall[4] (0.2 times). The geometric parameters of the fibre are listed in Table S1. The finite element calculations are performed using symmetric boundary condition and displacement loading.

Parameter	Description	Value	Description
H	Wall thickness	40 nm	[5]
L	Thickness of interface	30 nm	Micrographs
D	Diameter of the cell	605 nm	Micrographs

Table S1. Geometric parameters of the fibres.

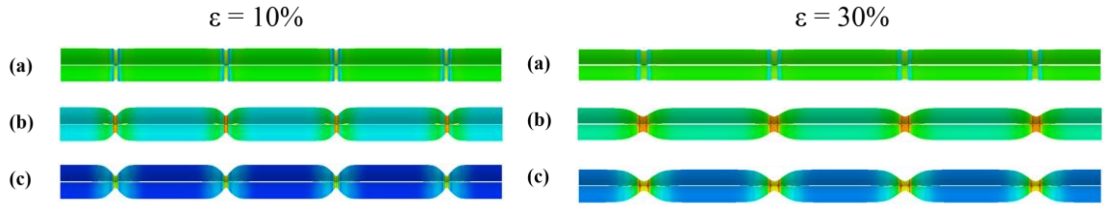

Figure S5. The front view of deformation behaviour under viscoelastic constitutive relation (10% and 30% strain). (a) Early stage in cell separation. **(b)** Middle stage in cell separation. **(c)** Late stage in cell separation.

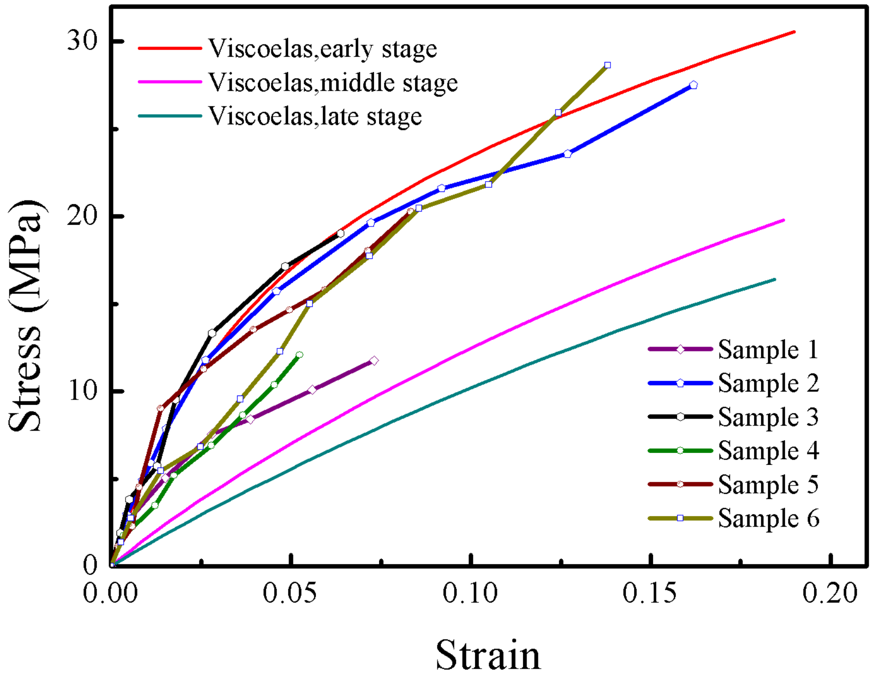

Figure S6. The calculated results (at three separation levels: early, middle and late stages in cell separation) compared with experimental results.

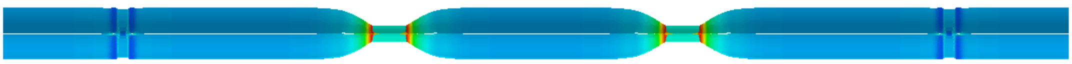

Figure S7. The front view of deformation behaviour of a fibre contains several septa at different separation levels under viscoelastic constitutive relation (50% strain).

The calculated results show that the periodic necking instability occurs at the positions of the septa (Fig. S5). The viscoelastic constitutive relation can well describe the experimental results, which all fall within the range of results calculated with FEA (Fig. S6). However, it is not sufficient to determine the deformation behaviour of the fibre dominated only by the viscoelastic mechanism from experimental stress–strain curves (Fig. 1e) at relatively high RH. The above-mentioned loading and unloading experiment shows definite viscoelastic deformation, so the periodic necking instability comes from structural changes (septa), and viscoelasticity dominates deformation behaviour of the multicellular fibres at high RH.

Because an individual fibre is connected by septa at various separation levels， as an example, we also study a fibre contains several septa at different separation levels (Fig. S4) under viscoelastic deformation. The necking mainly occurs at the positions of highly separated septa (Fig. S7). The type of deformation mode is affected by the separation levels of the neighbouring septa.

The above results show that septa play an important role in determining the evolution of fibre deformation and morphology.

---

1 Egan, A. J. F. & Vollmer, W. The physiology of bacterial cell division. *Ann. NY Acad. Sci.* **1277,** 8-28 (2013).

2 Turner, R. D., Vollmer, W. & Foster, S. J. Different walls for rods and balls: The diversity of peptidoglycan. *Mol. Microbiol*. **91,** 862-874 (2014).

3 Yang, Z. et al. Back-analysis of viscoelastic displacements in a soft rock road tunnel. *Int. J Rock Mech. Min*.**38,** 331-341 (2001).

4 Zhou, X. et al. Mechanical crack propagation drives millisecond daughter cell separation in *Staphylococcus Aureus*. *Science* **348**, 574-578 (2015).

5 Thwaites, J. J. & Mendelson, N. H. Biomechanics of bacterial walls: studies of bacterial thread made from *Bacillus subtilis*. *Proc. Natl Acad. Sci. USA* **82,** 2163-2167 (1985).
